# Supplementary figures and images for: A Novel SNP in EIF2AK4 Gene Is Associated with Thermal Tolerance Traits in Chinese Cattle
Source: Animals (Basel). 2019 Jun 19;9(6):375. doi: 10.3390/ani9060375 (PMC6617145; doi:10.3390/ani9060375)

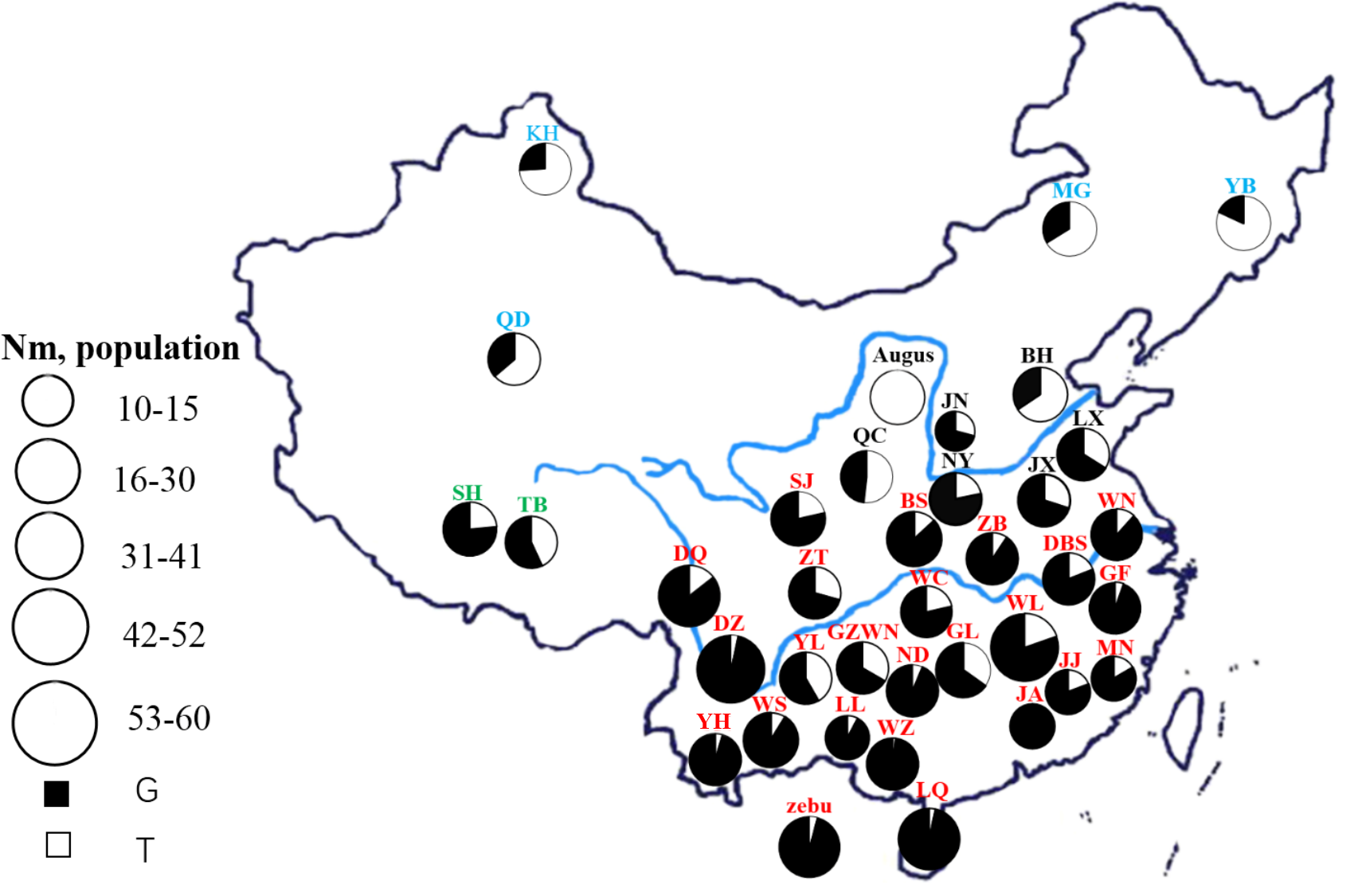

Supplement: Supplementary file 1 [file animals-09-00375-s001.zip › Supplementary files/Figure 1 Geographical distribution of EIF2AK4 variant among 35 Chinese indigenous cattle breeds as well as Angus and Indian zebu population. (2).tif]
